# PredGPI: a GPI-anchor predictor

**DOI:** 10.1186/1471-2105-9-392

**Published:** 2008-09-23

**Authors:** Andrea Pierleoni, Pier Luigi Martelli, Rita Casadio

**Affiliations:** 1 Biocomputing Group, Department of Biology, University of Bologna, Via Irnerio 42, 40126 Bologna, Italy

## Abstract

**Background:**

Several eukaryotic proteins associated to the extracellular leaflet of the plasma membrane carry a Glycosylphosphatidylinositol (GPI) anchor, which is linked to the C-terminal residue after a proteolytic cleavage occurring at the so called ω-site. Computational methods were developed to discriminate proteins that undergo this post-translational modification starting from their aminoacidic sequences. However more accurate methods are needed for a reliable annotation of whole proteomes.

**Results:**

Here we present PredGPI, a prediction method that, by coupling a Hidden Markov Model (HMM) and a Support Vector Machine (SVM), is able to efficiently predict both the presence of the GPI-anchor and the position of the ω-site. PredGPI is trained on a non-redundant dataset of experimentally characterized GPI-anchored proteins whose annotation was carefully checked in the literature.

**Conclusion:**

PredGPI outperforms all the other previously described methods and is able to correctly replicate the results of previously published high-throughput experiments. PredGPI reaches a lower rate of false positive predictions with respect to other available methods and it is therefore a costless, rapid and accurate method for screening whole proteomes.

## Background

In Eukaryotes, several integral membrane proteins can associate to the cell membrane by anchoring to its extracellular leaflet through Glycosylphosphatidylinositol (GPI) molecules.

All GPI-anchors have similar chemical structures, with minor differences among different species. The core of the anchor molecule comprises a sugar moiety and a phosphatidylinositol molecule, linked to two long-chain fatty acids. The sugar moiety is composed of a glucosamine, three mannose residues and one phosphoethanolamine that can form an amide bond with the C-terminal residue of a polypeptide (see [1] for a recent comprehensive review and references therein).

Free GPI-anchors are normally present in the plasma membrane and proteins are covalently bound to them after a post-translational modification occurring in the Endoplasmic Reticulum (ER) and comprising two steps: i) the cleavage in a specific position about 20–30 residues upstream the C-terminus (the so called ω-site); and ii) the removal of the carboxy-terminal portion (propeptide) of the protein precursor (proprotein).

Most of the GPI-anchored proteins are translocated to the plasma membrane, although there are some evidences of proteins residing in different compartments such as the ER or the Golgi apparatus [2]. After their export from the ER to the plasma membrane, mature proteins face the extracellular environment and perform many different functions by acting as enzymes, membrane receptors, surface antigens and adhesion molecules. Furthermore, being exposed on the external surface of cells, they can also be involved in signaling processes, immunomodulation and host-pathogen response. There are indications that GPI-anchored proteins reside preferentially in special patches of the plasma membrane enriched in cholesterol, sphingolipids and saturated phosphatidylcholine glycerids, known as lipid rafts, and that they are probably involved in recognition and signaling processes [3].

The GPI-anchor modification may be coupled with a transmembrane domain as it was experimentally proven that a transmembrane isoform of the human prion protein is endowed with a GPI-anchor [4], and that the protein BST-2 carries both a C-terminal GPI-anchor and a N-terminal transmembrane helix [5].

After the translocation to the external side of the membrane, some GPI-anchored proteins are released upon enzymatic cleavage of the anchor. Specifically in Fungi most of the GPI-anchored proteins are released from the plasma membrane and targeted towards the cell wall [6].

Due to their functional relevance, efforts are ongoing for discriminating how many GPI-anchored proteins can be expressed at the genome level.

Experimental determination of GPI-anchored proteins was carried out by means of phospholipase C or D solubilization. To our knowledge, to date, only three high throughput experiments have been set up for discriminating the GPI-proteome of two organisms, namely *Homo sapiens *and *Arabidopsis thaliana*; however they succeeded in finding only a tiny subset of all GPI-anchored proteins present in the proteome [7-9]. Even when a protein is detected as GPI-anchored, the experimental determination of the ω-site has to be done with low throughput procedures [1]. A reliable source of information, listing protein annotation along with experimental description, is the SwissProt database, which in release 53 contains 340 proteins that were experimentally proven to be GPI-anchored. Only 26 of these are endowed also with an experimentally characterized ω-site .

General features of GPI-anchored proteins are summarized in the following. Upon synthesis and upon recognition of a N-terminal signal peptide, proteins are targeted to the ER, where the C-terminal portion of the protein interacts with the transamidase complex by means of hydrophobic residues. This complex is responsible for the removal of the C-terminal domain (known as propeptide) and for the binding to a free GPI-anchor inserted into the internal leaflet of the ER membrane. Unfortunately no consensus sequence can be found to describe the localization of the ω-site. Nevertheless the C-terminal portion of the non-cleaved proteins can be roughly separated into different portions [10]:

• a linker region, comprising about 11 residues before the position ω-1; this is a region characterized by a low amount of predicted secondary structure;

• a region around the cleavage site, from ω-1 to ω+2, characterized by the presence of small side chain residues;

• a spacer region between the positions ω+3 and ω+9;

• a hydrophobic tail from ω+10 to the C-terminal end.

Typical residues in the few ω-sites experimentally annotated are: Cysteine, Aspartic acid, Glycine, Asparagine, and Serine. However, there is no stringent experimental evidence that other residues are prevented from acting as ω-site [1]. Moreover it was also experimentally proven that C-terminal regions of GPI-anchored proproteins can be exchanged between different organisms, without affecting the post-translational modification process [11].

Predictive methods are presently available in order to recognize GPI-anchored proteins and to determine the ω-site from the sole protein sequence and they differ on the computational method adopted to develop the algorithm. BIG-PI is based on a scoring function that takes into consideration the C-terminal features outlined above [10,12,13]; DGPI [14] in turn is based on a set of rules that are adopted to predict whether a protein is or is not GPI-anchored, searching for the above defined region in the C-terminal portion of the proprotein and recognizing the candidate ω-site. More recently GPI-SOM [15] makes use of self-organizing maps and signal peptide prediction with SignalP [16], achieving a better performance than the two previously described methods in discriminating GPI-anchored proteins. A very recent improvement was made by FragAnchor [17], a predictor able to recognize a high number of GPI-anchored proteins with few false positive errors. This is done by means of a two-step filtering procedure including a Neural Network (NN) and a Hidden Markov Model (HMM) that work in an independent way. However FragAnchor, unlike the other methods, is not able to assign a position for the ω-site. Another recent server, MemType-2L [18] is able to discriminate eight types of membrane proteins, including GPI-anchored proteins, by means of an ensemble of classifiers extracting information from position specific score matrixes computed after a PSI-Blast search. However, not even MemType-2L is able to predict the ω-site.

Here we describe PredGPI, a new method for discriminating GPI-anchored proteins and for determining the position of the ω-site. It makes use of a prediction system based on a Support Vector Machine (SVM) and a HMM that work in an integrated way. PredGPI outperforms all the other methods reaching a lower rate of false positive predictions and a consistent improvement in the coverage performance. Moreover the prediction of the ω-site localization is rather accurate, despite the scarcity of the data set. The good performance of the new method is due to the accurate choice of the training dataset and to the thorough selection of sequence features used as input to the methods.

## Methods

### The datasets

The dataset of GPI-anchored proteins was extracted from SwissProt 53, released on June 2007 [19]. Only experimental annotations were taken into consideration; proteins marked as 'fragment' and those whose annotations are reported as 'possible', 'probable', and 'by similarity', were excluded from the set. In order to avoid redundancy and to set up a correct cross-validation procedure, we filtered the downloaded sequences according to two criteria: the overall sequence identity and the E-value score obtained after the alignment of the 40-residue long C-terminal regions, which are likely to carry most of the information about the GPI anchoring.

Four datasets were therefrom collected:

1. *GPIω-Set*, which contains 26 proteins whose ω-sites are known. This set collects all the SwissProt entries that have an experimental annotation of the ω-site. All these proteins were checked in the literature to confirm the presence of the GPI-anchor and the location of the ω-site as reported in SwissProt. It has to be noticed that many entries, which were included in the training sets of previously developed tools, were erroneously annotated as experimental. In release 53 the annotations of the ω-sites of these proteins were revised and were indicated as "probable", "potential" or "by similarity". For this reason the revised entries were excluded from our training set. The 26 proteins of the *GPIω-Set *were clustered into 20 groups, each comprising the proteins with sequence identity greater than 30% or whose C-terminal tails align with an E-value lower than 0.001. Each set consists of one or two proteins and sequences in different sets do not share any detectable similarity. The 20 sets were used for performing a complete cross-validation of the HMM-based method for the prediction of the ω-site.

2. *GPI-Set*, which contains 145 proteins experimentally annotated in SwissProt as GPI-anchored; their ω-site in most cases is still undetermined. All the 145 proteins were checked in the literature to confirm the presence of the GPI-anchor. This set is reduced so that it does not contain pairs of proteins more than 30% identical or sharing similar C-terminal segments, setting an E-value threshold equal to 0.001 is fixed. This set comprises 8 non redundant sequences from the *GPIω-Set*.

3. *All-GPI-Set*, which contains all the proteins experimentally annotated as GPI-anchored in SwissProt, counting 340 examples. This set comprises both the *GPIω-Set *and the *GPI-Set*.

4. *Non-GPI-Set*, comprising 10,630 proteins chains less than 30% identical and not annotated as GPI-anchored.

*Non-GPI-Set *and *GPI-Set *were used for training the SVM-based method when discriminating GPI-anchored proteins. Prediction performances were evaluated with a complete jack-knife procedure. Thus, it is very important to consider only sequences sharing low identity (in our case, less than 30%).

It is worth noticing that MemType-2L [18] and GPI-SOM [15] adopt higher identity thresholds, equal to 80% and 50%, respectively, when testing with jack-knife or other cross validation procedures. The descriptions of FragAnchor [17] and DGPI [14] do not take into consideration the homology issue, while BigPI [10] was evaluated with a jack-knife test on a non-homologous data set, without declaring which identity threshold was considered.

Our stringent definitions for reducing the redundancy in the collected sets assure no overfitting on the training data, even when the training set is very small as in the case of GPI-anchored proteins with known cleavage sites.

### HMM model of the ω-site

The main features characterizing the C-terminal portion of GPI-anchored proteins, where the ω-site is located, can be cast in a hidden Markov model (HMM), a graphical model composed of states, each one representing a position along the sequence. The peculiar residue composition of different regions of the sequence are described by means of the emission probabilities assigned to each state; the states are connected by transition probabilities [20].

**Figure 1:**
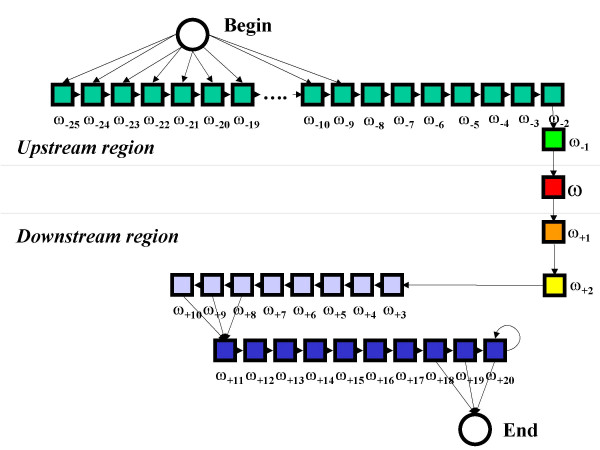
**The HMM model of the ω-site**. Different colors represent different emission probability sets. ω-site is represented in red. Surrounding residues are colored in green, orange and yellow. The preceding region is represented in dark green. The spacer and the C terminal hydrophobic regions are depicted in violet and blue, respectively. The total number of independent trainable parameters is 147.

In particular the model depicted in Figure 1 is designed to describe the 40-residue C-terminal segment of the GPI-anchored proteins. It contains 46 states centered on the state describing the ω-site. The states filled with the same color share the same emission parameters so that the model describes different zones with different residue compositions. The ω-site, the residue upstream and the two residues downstream are described with independent emission probabilities. The regions upstream and downstream the ω-site neighbors are described with one and two sets of emission probabilities, respectively. Two extra states serve for beginning and ending the process and do not emit any letter. The topology of the transitions describes C-terminal cleaved propeptides (the portions following the ω-sites) longer than 16 residues and models their experimental length distribution. The model was trained for recognizing the ω-sites starting from the C-terminal sequences of the proteins included in the *GPIω-Set*. Single sequence coding and labeled Baum-Welch training were adopted, using the three labels: Upstream, ω, and Downstream. A complete cross validation was performed, using all the 26 sequences with experimentally known ω-site divided into 20 sets: 19 sets were used for training and the remaining for testing. Since the 20 sets share low identity, this procedure gives a correct estimate of the performance and it is not biased by the homology of the sequences. Due to the scarcity of the known examples, pseudocounts were used when updating the emission parameters to increase the generalization performances. The posterior Viterbi algorithm was used for decoding [21]. Given a sequence, this algorithm optimally aligns it to a given model, maximizing the *a posteriori *probability for the emission and complying with the topological constraints of the model. The predicted ω-site is the residue that is aligned with the ω-site state. The emission probability of the sequence is also computed and used as input to the SVM discriminator described in the next section. A conservative HMM was also trained, without adding any pseudocount during the training procedure. In this way the prediction is more constrained and in particular it allows as ω-sites only the residues that are observed in experimentally annotated sequences, namely Cysteine, Aspartic acid, Glycine, Asparagine, and Serine. However, as we observed in the Introduction, there is no stringent evidence for excluding other residues.

### SVM based discriminator for GPI-anchored proteins

Support Vector Machines (SVMs), first introduced by Cortes and Vapnik [22], and are able to optimally discriminate between two classes: inputs are coded with a numerical vector and then mapped into a *h*-dimensional space *H*, by means of a kernel function. SVMs are algorithms able to draw a (*h*-1)-dimensional hyperplane in the space *H*, for discriminating the two classes.

For discriminating between GPI-anchored and non-GPI-anchored proteins, we used the SVM-light implementation for SVMs that is freely available at . The input combines the probability output of the HMM model described in the former section with information derived from the whole sequence, the carboxy-terminal region (C-ter), and the amino-terminal region (N-ter). The feature vector for each residue sequence is composed of 83 elements and describes the overall composition of the sequence, the features of the N-terminal regions (N-ter), comprising the signal peptide, and the features of the C-terminal regions (C-ter), containing the cleaved GPI-anchor signal. More specifically the 83-valued input vector consists of:

• 20 values ranging from 0 to 1, reporting the residue composition of the whole sequence;

• 20 values ranging from 0 to 1, reporting the residue composition of the last 40 C-ter residues;

• 20 values ranging from 0 to 1, reporting the residue composition of the last 20 C-ter residues;

• 20 values ranging from 0 to 1, reporting the residue composition of the first 20 N-ter residues;

• one value ranging from -1 and 1, reporting the average Kyte-Doolittle hydrophobicity of the last 20 C-ter residues; the original Kyte-Doolittle scale, ranging from -4.5 associated to Arginine and +4.5 associated to Isoleucine, was linearly rescaled between -1 and 1 [23];

• one value reporting the average Kyte-Dollittle hydrophobicity of the first 20 N-ter residues; the original Kyte-Doolittle scale was rescaled as described above;

• one value reporting the negative logarithm of probability computed by the HMM-based ω-site predictor.

A complete jack-knife validation procedure was performed considering the 145 positive examples and the 10,630 negative examples included in *GPI-Set *and *Non-GPI-Set*, respectively. It is worth to stress that the performances evaluated with the jack-knife procedure are reliable since all the sequences are less than 30% identical.

The Radial Basis Function (RBF) Kernel was adopted to map the feature vectors. After an extended search in the parameter space the best SVM separation, as measured by the maximum MCC index (see next section), was obtained setting the parameters C = 6, and γ = 3. For sake of rapidity, the search in the parameter space was performed with a 10-fold cross validation procedure. The 10 cross-validation sets were compiled randomly, and contain all the sequences in *GPI-Set *and *Non-GPI-Set*. Since all the considered sequences share low identity, this procedure does not bias the results.

For each example, SVM-light reports the distance of the feature vector from the discriminating hyperplane. On the basis of these distances different thresholds can be fixed for tuning the false positive and false negative rates.

### Evaluation and comparison with other predictors

BIG-PI [10,12,13], GPI-SOM [15], FragAnchor [17] and MemType-2L [18] web server predictors were interrogated to test our datasets, while DGPI [14] was run locally with the last free available distribution. When testing BIG-PI, which implements different parameterizations for the different kingdoms, the suitable predictor was used for each protein.

Four parameters were used to evaluate the prediction performances. We indicated with TP and TN the number of True Positive and True Negative predictions, respectively, and with FP and FN the number of False Positive and False Negative predictions, respectively.

The Coverage, or true positive rate, was calculated as the number of proteins correctly predicted as GPI-anchored over the total number of positive examples.

(1) $$Cov=\frac{TP}{TP+FN}$$

The Accuracy value corresponds to the number of proteins correctly predicted as GPI-anchored over the total number of protein predicted as GPI-anchored.

(2) $$Acc=\frac{TP}{TP+FP}$$

The false positive rate corresponds to the number of protein predicted as GPI-anchored but annotated as negative examples over the total number of negative examples.

The Matthews Correlation Coefficient was calculated as:

(3) $$MCC=\frac{TP\cdot TN-FP\cdot FN}{\sqrt{\left(TP+FP)(TP+FN)(TN+FP)(TN+FN\right)}}$$

A thorough explanation of the purposes of these indexes can be found in [24].

### Assessment of the most relevant features

In order to evaluate the relevance of each feature in the prediction process, a feature elimination approach was adopted. 83 different SVM discriminators were trained, each one using only 82 features. A complete jack-knife procedure was adopted, considering the 10,775 non homologous examples comprised in *GPI-Set *and *Non-GPI-Set*. The relevance of a feature is measured by means of the decrease in the performance of the SVM that do not consider that feature: the higher is the decrease, the most relevant is the information conveyed by the missing feature. The predictive score was evaluated in terms of MCC. For each feature i, the variation in MCC can be computed as follow:

(4) Δ*MCC*(*i*) = *MCC*(*i*) - *MCC*

where MCC(i) is the correlation coefficient reached by the SVM lacking the feature i and MCC is the correlation coefficient reached by the SVM using all the features.

## Results and discussion

### Prediction performances: discrimination of GPI-anchored proteins

**Figure 2:**
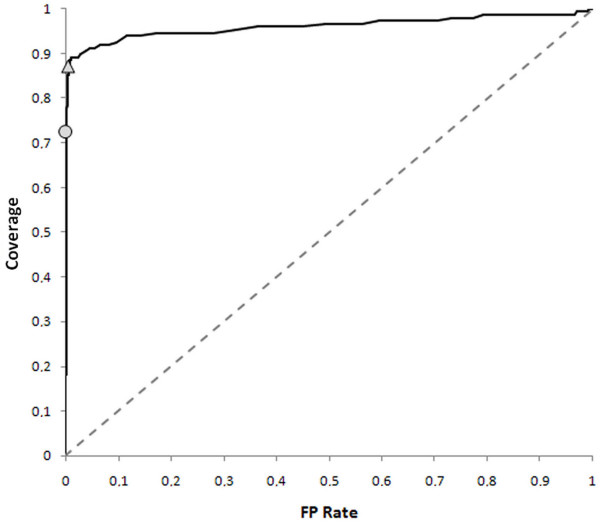
**The ROC curve of PredGPI**. The ROC curve of PredGPI is shown as a continuous line. The dashed line is referred to a random guess. Two points are shown over the ROC curve: the circle indicates a false positive rate of 0.15%, while the triangle indicates a false positive rate of 0.5%. The curve was computed using the 145 positive examples and the 10,630 negative examples in *GPI-Set *and *Non-GPI-Set*, respectively. See text for details in the Methods section.

The performances of the discrimination of the GPI-anchored proteins were computed with a complete jack-knife procedure, and are described by the ROC curve depicted in Figure 2: the Coverage is plotted versus the rate of false positives when varying the discrimination threshold, which is the distance from the separating hyperplane. It is evident that the performance of the method is very different from that of a random guess, which would give origin to a linear plot on the main diagonal line.

Two thresholds have been chosen and are represented in the plot. The first one indicates a false positive rate as low as 0.15% corresponding to a coverage as high as 77% and to the maximum Matthews Correlation Coefficient (MCC = 0.82). The second threshold allows increasing the coverage up to 89%, with a false positive rate still as low as 0.5%.

The performances obtained with PredGPI and with the other publicly available predictors, namely BIG-PI [10], DGPI [14], GPI-SOM [15], FragAnchor [17], and MemType-2L [18] are listed in Table 1. All the predictors were evaluated on the 145 positive examples and 10,630 negative examples contained in *GPI-Set *and *Non-GPI-Set*, respectively. It is worth noticing that in the case of PredGPI the results were obtained with a complete jack-knife procedure on a set of sequences sharing low identity, but most of the proteins used in the evaluation were included in the training process of the other predictors. Unfortunately, due to the lack of new release of experimentally annotated sequences we are unable to build a dataset for a completely blind test.

**Table 1:** Comparison between PredGPI and other available predictors

Predictor	TP	FP	Cov (%)	Acc (%)	FP rate (%)	MCC
PredGPI	112	15	77.2	88.2	0.14	0.823
FragAnchor	102	37	70.3	73.4	0.35	0.725
BIG-PI	79	33	53.4	70.5	0.31	0.609
DGPI	117	250	79.1	31.9	2.35	0.492
GPI-SOM	126	182	85.1	40.9	1.7	0.583
MemType-2L (Shangai server*)	74	189	51.0	28.1	1.8	0.368
MemType-2L (Harvard server*) **	≤ 107	≥ 60	≤ 73.8	≤ 64.1	≥ 0.56	≤ 0.683

Performances are evaluated on 145 positive and 10,630 negative examples contained in *GPI-Set *and *Non-GPI-Set*, respectively. PredGPI performances were evaluated using the jack-knife procedure. It's worth noticing that many of the tested proteins may have been used for the training of other predictors.

Abbreviations: TP = True Positives, FP = False Positives; the number of sequences is listed; Cov = Coverage, Acc = Accuracy, FP rate = False Positives over the total number of negative examples, MCC = Matthews Correlation Coefficient.

*MemType-2L is available in two versions: the Shangai server  and the Harvard server . For sake of completeness we used both.

** The Harvard server of MemType-2L gave an answer only for 143 out of the 145 positive examples comprised in *GPI-Set*. 105 sequences are correctly predicted in this set. Moreover the server gave an answer only for 4,265 out of the 10,630 negative examples comprised in *Non-GPI-Set*. The number of mispredictions in this set is equal to 60. The limits to the indexes scoring the performance of this server are computed in the best case, which is by considering all the non predicted proteins as correctly predicted.

BIG-PI is the first publicly released method for GPI-anchor prediction, and the predictions are made by four kingdom-specific predictors [10,12,13]. This method is able to recognize only half of the submitted GPI-anchored proteins while maintaining a false positive rate as low as 0.3%. More recently two new predictors were implemented, DGPI [14] and GPI-SOM [15], which are able to recognize a larger number of GPI-anchored proteins but the false positive rates of these methods are very high, equal to 2.3% and 1.7% respectively. These values have to be compared with the number of GPI-anchored proteins in a proteome, which can be estimated to be around 0.5–1% [10]. FragAnchor [17] is a very recent predictor that is able to achieve a coverage value of 70%, while maintaining the same false positive rate of BIG-PI. Concerning Mem-Type2L [18], two different servers are available, at the Shangai University and at the Harvard University, respectively. They are declared to be mirrors of the same method. However they perform very differently, so we evaluated both of them. The former scores with low Coverage (51%) and a quite high false positive rate. Performances of the Harvard server could be only partially evaluated, since it did not give answer in 6,357 cases out of the 10,775 tested proteins. Even when evaluated in the best case, which is assuming that all the non predicted proteins are correctly predicted, it scores with a Correlation index lower than that of FragAnchor, since the increase in Accuracy is compensated by the increase in false positive rate. Our method is able to greatly outperform all the other predictors in both accuracy and correlation coefficient. Even halving the false positive rate with respect to BIG-PI and FragAnchor, PredGPI is able to achieve a 77% coverage value and a correlation score of 0.82. When considering a less stringent threshold for the false positive rate (0.5%), PredGPI is able to correctly identify 89% of GPI-anchored proteins. This coverage value is higher than those obtained by DGPI and GPI-SOM, while maintaining again a much lower false positive rate.

The released version of PredGPI, trained with the complete datasets, was used to predict all the proteins experimentally annotated as GPI-anchored in SwissProt (see Table 2).

**Table 2:** Coverage on all the experimentally annotated proteins in SwissProt

Predictor	TP	Cov
PredGPI	301	88.5
FragAnchor	286	84.1
BIG-PI	189	55.6
DGPI	267	78.5
GPI-SOM	278	83.8
MemType-2L (Shangai server*)	147	43.2
MemType-2L (Harvard server*) **	≤ 293	≤ 86.1

The testing dataset comprises all the 340 GPI-anchored proteins experimentally annotated in SwissProt and contained in *All-GPI-Set *Abbreviations: TP = True Positives; Cov = Coverage.

* See legend to Table 1.

** The Harvard server of MemType-2L gave an answer only for 309 out of the 340 positive examples comprised in *All-GPI-Set*. 262 sequences are correctly predicted in this set. The limits to the indexes scoring the performance of this server are computed in the best case, namely by considering all the non predicted proteins as correctly predicted.

A total of 340 proteins were screened from the *All-GPI-Set *(see Methods). PredGPI correctly recalls 301 positive examples (corresponding to 88.5%) as GPI-anchored when using the threshold corresponding to a false positive prediction rate lower than 0.1%. By setting the less stringent threshold (FP rate = 0.5%) PredGPI is able to recall up to 93% of the experimentally annotated sequences. Without considering the incomplete results obtained by the Harvard server of MemType-2L, the other methods range from the 286 (84.1%) proteins correctly predicted by FragAnchor, to the 147 (43.2%) proteins predicted by the Shangai server of Memtype-2L.

### Prediction performances: prediction of the ω-site

Concerning the prediction of the localization of the ω-site, the HMM correctly identifies all but five cleavage sites, when evaluated in cross-validation. In three cases the predicted ω-site is only a position apart with respect to the real site (Table 3). Only for sequences FOLR1_HUMAN and ACES_TORMA, the difference between the predicted and the real ω-sites is equal to 6 and 5 residues respectively. When a predictor trained on all the available proteins is used, only two ω-sites are mispredicted, both of them by just a position. The results are compared with those obtained with the other methods.

**Table 3:** Performance for the prediction of the ω-site

Predictor	BigPI	DGPI	GPI-SOM	PredGPI (cv*)	PredGPI (non cv**)
Correctly predicted proteins	23	16	15	21	24
Proteins wrongly predicted by one position	1	4	2	3	2
Proteins wrongly predicted by more than one position	1	5	7	2	0
Proteins predicted as non GPI-anchored	1	1	2	0	0

The test set comprises 26 sequences with experimentally annotated ω-site (GPIω-Set). The number of sequences is listed.

*cv = results obtained with a 20-fold cross validation prediction;

**non cv = results obtained with a predictor trained on all the 26 sequences.

PredGPI outperforms ω-site prediction performances of both DGPI and GPI-SOM that are able to correctly predict only 17 and 15 sites, respectively. The BIG-PI predictions achieve a performance comparable to that of PredGPI, being able to correctly annotate 23 out of 26 sites: one site is mispredicted by one position and a second site by more than five positions. One last protein was not predicted as GPI-anchored. Again, due to the scarcity of the dataset, most of the proteins used in this test are likely to be included in the training procedure of other methods. It is worth noticing that our method is the only one able to assign all the proteins of the *GPIω-Set *as GPI-anchored.

The ω-sites in the proteins of the *GPIω-Set *are formed only by Cysteine, Aspartic acid, Glycine, Asparagine, and Serine. Since no evidence has been reported about the exclusiveness of these residues, a flexible HMM was trained that allows other residues as ω-site. When predicting the set of all the 340 experimentally known GPI-anchored proteins, 77% of the predicted ω-sites are formed by the five above listed typical residues. A more restrictive HMM is available in the PredGPI web server to predict the ω-sites without allowing non-typical residues.

### Evaluation on data derived from high-throughput experiments

Up to date, three large scale experiments have been carried out to find GPI-anchored proteins in *Homo sapiens *and *Arabidopsis thaliana *by means of phospholipase C or D digestion and a subsequent two-phase partitioning. These techniques are not able to detect all of the GPI-anchored proteins expressed by the two considered organisms. For this reason the proteins detected with these procedures can be used just to evaluate the false negative rate, but not to estimate the false positive rate. In 2003, Borner *et al*. performed a high-throughput experiment to identify GPI-anchored proteins from the callus of *Arabidopsis thaliana*; a negative control, not treated with phospholipase C, was used to reduce false positive annotations [7]. With this method, Borner *et al*. isolated 30 experimentally verified GPI-anchored proteins. With a different approach Elortza *et al*. isolated, after digestion with phospholipase, 42 chains in *Arabidopsis *callus, 35 of which were validated as *bona fide *GPI-anchored proteins after the consensus prediction with BIG-PI, DGPI and GPI-SOM [8].

When considering the 34,804 protein sequences encoded by the *Arabidopsis *genome (Integr8 v.75), PredGPI predicts 435 GPI-anchored proteins. This set comprises all the 30 sequences determined by Borner *et al*. [7] and 35 of the 42 chains isolated by Elortza *et al*. [8] after digestion. 34 sequences out of 35 are in agreement with the consensus prediction considered by the authors. The protein RETOL_ARATH (TAIR: At4g20830), which is predicted as GPI-anchored by the consensus method and not by PredGPI, has been previously reported as a major contaminant in high throughput experiments [9]. This fact strengthens the PredGPI prediction. On the other hand PredGPI identifies the protein Q9T0A9_ARATH (TAIR: At4g23950) as GPI-anchored.

With the same procedure Elortza *et al*. [8] isolated after digestion 42 chains from the HeLa human cell line, 11 of which were considered *bona fide *GPI-anchored proteins by the consensus predictive method.

When predicting the 48,400 protein sequences of the Human genome (ENSEMBL 48) with PredGPI, 541 are discriminated as GPI-anchored. This set comprises 11 out of the 42 proteins experimentally isolated after digestion. The agreement between PredGPI and the *bona fide *prediction amounts to 10 sequences.

A novel protein is predicted as GPI-anchored: CAC2D_HUMAN (L-type calcium channel subunit alpha-2 alpha 2/delta subunit precursor). This protein is currently annotated to be endowed with a monotopic C-terminal transmembrane domain. However there is no experimental evidence for the presence of a membrane spanning segment [25]. Following our prediction, the C-terminal hydrophobic domain is cleaved and the protein is GPI anchored at Gly 1060.

These tests, aimed to evaluate the false negative rate on proteins with experimental validation, prove that PredGPI is able to annotate GPI-anchored proteins with the same accuracy of an approach based on the coupling among different predictors and experimental procedures, and to correctly annotate almost all of the experimentally annotated GPI-anchored proteins. Since the use of PredGPI is not restricted by experimental constraints it can be applied in a few minutes to an entire proteome to obtain costless, high quality data. The lists of proteins predicted as GPI-anchored by PredGPI in *Homo sapiens *and *Arabidopsis thaliana *are available at .

### Analysis of the most relevant features

We evaluated the relevance of each one of the 83 features used in the prediction by measuring the decrease in performance when a SVM was trained without using that feature. Table 4 lists the 10 most relevant features according to the decrease in MCC with respect to 0.823, the value of MCC when all the features are included. For each feature, the third column in Table 4 indicates whether the average of the considered feature is higher in GPI- or non GPI-anchored proteins, as measured considering the non homologous examples contained in the *GPI-Set *and *Non-GPI-Set*.

**Table 4:** Most relevant features as evaluated by MCC decrease upon feature elimination

Feature	ΔMCC	Higher in
Average KD hydrophobicity of 20 C-ter residues	-0.021	GPI
Frequency of Ser in 40 C-ter residues	-0.020	GPI
Frequency of Leu in 40 C-ter residues	-0.018	GPI
Frequency of Gly in 20 C-ter residues	-0.016	GPI
Frequency of Asn in 20 N-ter residues	-0.016	Non GPI
Frequency of Asn in whole sequence	-0.015	GPI
Frequency of Gln in 20 N-ter residues	-0.015	Non GPI
Frequency of Leu in 20 N-ter residues	-0.015	GPI
Frequency of Thr in whole sequence	-0.015	GPI
Frequency of Ala in 20 N-ter residues	-0.015	GPI

Only features leading to a Δ MCC lower than -0.015 were listed. The third column indicates whether the considered feature has higher average value in GPI- or in non GPI-anchored proteins, as computed considering the sequences included in *GPI-Set *and *Non-GPI-Set*.

The highest decrease amounts to 0.021 and it is related to the average hydrophobicity value of the last 20 C-terminal residues, as measured with the Kyte-Doolittle scale. This accounts for the presence of a highly hydrophobic tail in the C-terminus of all the GPI-anchored proteins. The next highest decrease amounts to 0.020 and it is related to the frequency of Serine in the last 40 C-terminal residues. This agrees with the observation that Serine is the most frequent residue found in the experimentally detected ω-sites; moreover short Serine-rich repeats are frequently present in the cleaved propeptide [1]. Two more residues in the C-terminal regions enhance the discriminative power of the SVM: Leucine and Glycine, whose significancy is justified by the hydrophobic character of the cleaved propeptide. The compositions in Asparagine, Glutamine, Leucine, and Alanine in the 20 residue N-terminal regions are particularly important for the discrimination. Indeed all the reported GPI-anchored proteins are endowed with a N-terminal signal peptide, whose composition is rich in hydrophobic residues and in particular in Alanine and Leucine. By comparing the residue composition between the 20-residue N-terminal regions of GPI- and non GPI-anchored proteins, the major differences are due to the composition in Asparagine and Glutamine, mostly present in non-GPI anchored proteins, and in Alanine and Leucine, particularly abundant in N-terminal regions of GPI-anchored proteins (data not shown). The two other features that emerged with the feature elimination procedure are the overall composition in Threonine and Asparagine. Both residues are more abundant in GPI-anchored proteins; in particular, comparing the compositions of proteins in our data sets, Threonine emerges as the residue endowed with the most relevant difference between GPI- and non GPI-anchored proteins.

On the overall, the feature elimination procedure highlighted compositional features that in most cases are confirmed as the most discriminative by statistical analysis (Alanine, Leucine, Asparagine, and Glutamine at the N-terminus, Threonine in the whole sequence) or that are supported by previous findings (Serine and hydrophobic residues at the C-terminus).

When the other features are taken into account, the elimination of the average Kyte-Doolittle hydrophobicity of the N-terminal 20-residue regions results in ΔMCC equal to -0.008, while the elimination of the feature reporting the probability computed by the HMM results in a MCC decrease equal to -0.004.

In evaluating these results it has to be considered that SVMs with RBF kernels are highly non-linear machine learning tools that combine features in a very complex way, so that it is difficult to infer the relevance of each feature. In particular, the feature elimination procedure estimates the information that each single feature adds to the others. Our results indicate that all the 83 considered features are to different extents relevant and we used all of them in the released predictor.

### PredGPI prediction server

The PredGPI prediction server is freely available at: .

For every submitted protein the prediction system gives the position of the most probable ω-site together with a measure of the probability of the presence of the GPI-anchor expressed as the *specificity *index (defined as: 1 – FP rate). For each prediction we used the distance on the discriminating hyperplane as computed by the SVM for evaluating the specificity on the basis of the thresholds derived in the training phase (see ROC curve in Fig 2). When the specificity is higher than 99.9% the prediction is flagged as "GPI-anchored: highly probable"; when the specificity ranges from 99.9% to 99.5% the prediction is flagged as "GPI-anchored: probable"; when the specificity ranges between 99.5% and 99.0% the prediction if flagged as "GPI-anchored: lowly probable". The user can choose between the conservative and the non-conservative HMM to predict the ω-site position.

All datasets are available on the web server.

## Conclusion

Here we presented PredGPI, a new method for predicting GPI-anchored proteins. The system is able to give high accuracy predictions that discriminate up to 89% of the known GPI-anchored proteins with a false positive rate equal to 0.15%. The coverage increases up to 93% when setting a threshold corresponding to a false positive rate equal to 0.5%. PredGPI outperforms all the other currently available prediction methods, being more accurate and able to predict a higher amount of proteins.

PredGPI is also a reliable method for the annotation of ω-sites proving to correctly predict 21 out of 26 annotated ω-sites, and missing only three by just by one position.

## Authors' contributions

AP built the datasets, implemented the SVM, set up the web server and wrote the manuscript; PLM implemented the HMM and wrote the manuscript; RC supervised the design of the research and wrote the manuscript. All authors read and approved the final manuscript.
